# A Study on the Quality of Life and Economic Burden Among People Living With HIV/AIDS and Attending a Tertiary Care Hospital in Jharkhand, India

**DOI:** 10.7759/cureus.88640

**Published:** 2025-07-24

**Authors:** Mithilesh Kumar, Nisha Murmu, Anit Kujur, Shashi Singh, Dewesh Kumar, Vidya Sagar

**Affiliations:** 1 Community Medicine, Rajendra Institute of Medical Sciences, Ranchi, IND; 2 Community Medicine and Family Medicine, All India Institute of Medical Sciences (AIIMS) Bhubaneswar, Bhubaneswar, IND; 3 Preventive Medicine, Rajendra Institute of Medical Sciences, Ranchi, IND

**Keywords:** antiretroviral treatment centre, economic burden, hiv aids, plwha, quality of life (qol)

## Abstract

Background

AIDS, a potentially life-threatening disease caused by a retrovirus known as human immunodeficiency virus (HIV), has evolved from a mysterious illness to a global pandemic, affecting tens of millions of people. HIV/AIDS poses an increasing threat to the health of the population and causes further socioeconomic loss for people, communities and governments in many countries and drastically affects the quality and standard of life. Very few studies have used the World Health Organization Quality of Life HIV Brief Scale (WHOQOL-HIV BREF) instrument in people living with HIV and AIDS (PLWHA). The study aims to assess the quality of life (QOL) and economic burden of PLWHA and to determine various factors affecting their QOL.

Methods

The study was an institution-based cross-sectional study at an Anti-Retroviral Therapy Centre of a tertiary care hospital in the state of Jharkhand in India from June 2018 to November 2020. The study population consists of HIV/AIDS patients aged 18 years or older and who had been diagnosed as HIV positive and were on antiretroviral therapy (ART) for more than a month. Systematic random sampling was used to recruit 216 patients for this study. Data collected using the semi-structured questionnaire, WHOQOL-100 and WHOQOLHIV-BREF for assessing QOL and a self-designed, semi-structured and pretested proforma was used to assess the economic burden of PLWHA. The data were analysed by using the IBM Statistical Package for Social Science (SPSS) version 20.0 (IBM Corp, Armonk, NY, USA). Frequency tables were generated to see the distribution of variables. Student’s t-distribution test was performed to assess the association between the mean score of QOL and dichotomous variables. One-way analysis of variance (ANOVA) was used for the variables with multiple categories. Correlation and multiple linear regression were implemented to see the association between economic burden and QOL. For all statistical analyses, a p-value <0.05 was considered significant.

Results

The mean age (±standard deviation (SD)) of 216 patients was found to be 40.48 (±11.87) years. In this study, participants reported a QOL score mean (±SD) of 55.48 (±8.006) for the physical domain, 64.87 (±6.920) for the psychological domain, 54.95 (±5.308) for the level of independence domain, 60.67 (±5.586) for the social relationship domain, 67.98 (±3.940) for the environmental domain and 49.42 (±5.202) for the spiritual/religious/personal beliefs (SRPB) domain. The total QOL score was found to be 57.79 (±3.248). The total mean economic burden was assessed to be INR 36,182.94 (±5606.98) per month. The study showed a significant association between the independent variables - total medical costs (p-value=0.022), loss of wages (p-value=0.005) and total economic burden (p-value =0.004) - and the total QOL scores.

Conclusion

Economic burden plays an important part in affecting the lives of people with HIV/AIDS. There is a need for financial intervention and rehabilitation programs catering to the needs of PLWHA, which will not only give them a sense of security but also lead them to have a community where they will feel welcome and be satisfied with their life.

## Introduction

AIDS, historically referred to as the "slim disease," has evolved from a mysterious illness into a global pandemic impacting tens of millions of people worldwide. It is a potentially life-threatening condition caused by the human immunodeficiency virus (HIV), a retrovirus that attacks and progressively weakens the immune system. Once infected, the virus remains in the body for life, making HIV/AIDS a persistent global health challenge that affects both industrialized and developing nations. The impact of HIV/AIDS extends beyond health, resulting in substantial socio-economic consequences for individuals, communities, and governments globally [1].

By the end of 2024, approximately 40.8 million people were living with HIV worldwide, including around 1.4 million children younger than 15 years. In the same year, about 1.3 million people were newly infected, and roughly 630,000 people died from AIDS-related illnesses [2]. While HIV/AIDS remains potentially fatal if left untreated, the advent and widespread availability of antiretroviral therapy (ART) have dramatically improved the prognosis for people living with HIV. As of 2024, over 31.6 million individuals were accessing ART globally, enabling many to manage HIV as a chronic condition and lead longer, healthier lives [2,3].

The World Health Organization (WHO) has defined quality of life (QOL) as individuals’ perceptions of their position in life in the context of the culture and value systems in which they live and in relation to their goals, standards, expectations and concerns. This definition highlights the importance of an overall subjective feeling of well-being pertaining to aspects of morale, happiness and satisfaction. It usually refers to the degree to which a person's life is desirable or undesirable [4]. Several instruments for measuring QOL have been developed and described, for example the World Health Organization Quality of Life-100 (WHOQOL-100), which is a cross-cultural instrument initiated and developed by the World Health Organization. However, very few studies have applied the World Health Organization Quality of Life HIV Brief Scale (WHOQOL-HIV BREF) instrument for people living with HIV and AIDS (PLWHA). This scale has six domains, namely, physical, psychological, level of independence, social relationships, environment and spiritual/religion/personal beliefs [5,6].

HIV/AIDS drastically affects the quality and standard of life. The opportunistic infection, side-effects of ART and other symptoms lead to psychological and physical problems in the individuals [7]. The factors affecting the QOL of an HIV patient are multidimensional [8]. The factors related to disease include CD4 count, opportunistic infections and HIV stage, among others. The emotional factors include the distress of the progression of disease, depression, the social stigma and discrimination, which create an emotional toll on the patients [9-12]. Additionally, socio-demographic attributes such as age, gender, education and employment also resulted in decreased QOL [13-15].

India remains the country with the third largest HIV epidemic in the world. In 2023, HIV prevalence among adults (age 15-49 years) was estimated at 0.20%. While this prevalence is low compared to many other middle-income nations, India’s large population - over 1.4 billion - means that approximately 2.5 million people were living with HIV in 2023. Recent years have seen significant progress; the number of new HIV infections in 2023 was estimated at about 66,400, representing a 44% reduction since 2010 [16,17]. AIDS-related deaths have also declined sharply, with an estimated 38,900 deaths from AIDS-related illnesses in 2023 - a 76% reduction since 2010. These figures highlight the continued success of prevention and treatment programs [18]. In terms of treatment coverage and the care cascade, 95% of people living with HIV in India now know their status, 88% of those diagnosed are on ART, and 85% of people on ART are virally suppressed as of 2023. These advances bring India close to the UNAIDS 95-95-95 targets for HIV care and viral suppression [19].

According to India HIV Estimates 2023, Jharkhand is estimated to have approximately 23,794 people living with HIV/AIDS, a notable decrease from the 34,386 cases reported in 2017. Between 2007 and 2017, the state experienced a 59% increase in total HIV/AIDS cases; however, recent years show a relative stabilization or decline in the epidemic’s burden, partly influenced by a nearly 50% reduction in HIV testing between 2023 and 2024, which may have affected case detection and reporting accuracy. Annual new HIV infections in 2023 were approximately 744, while the total number of AIDS-related deaths was 194 [18].

No previous study has been done on QOL and economic burden in Jharkhand and there is still a dearth of research to find the impact of economic burden on QOL in India. The purpose of this study is to assess the QOL of and economic burden on PLWHA and to find out various factors affecting the QOL to provide current information to policymakers and program managers about the burden on PLHIV both economically and socially.

## Materials and methods

The study used was an institution-based cross-sectional study and the study population were HIV/AIDS patients aged 18 years or older and who had been HIV positive and were on ART for more than a month prior to the study were included in the study. The study was conducted from June 2018 to November 2020 at an ART Centre of a tertiary care hospital of Jharkhand, namely Rajendra Institute of Medical Sciences, Ranchi.

The sampling method used in the study was systematic random sampling. A minimum of 300 patients who met the inclusion criteria were supposed to be taken as the study sample (a minimum of 25 patients to be recruited per month) as per the record from ART Centre, that is, 25 patients per month * 12 months of data collection = 300 patients. The study subjects were selected according to the registration number. The systematic sampling interval (k) was calculated as 4851/300≈16. A random number selected between 1 and 16 was 10. This registration number was taken as the first study patient and then every 16th patient was taken into the study, like 10, 26, 42, 58, 74, 90, etc. till the total of 300 subjects were interviewed. Data collection was started late from June 2019, and it could be continued only till February 2020 after which it could not be done due to the COVID-19 pandemic. Thus, since that time only 216 HIV/AIDs patients were interviewed, the sample size was taken as 216.

The questionnaire used to assess the QOL in this study was developed by adapting items from two established instruments - the WHOQOL-100 and the WHOQOL-HIV BREF. After a careful review, only those questions deemed most relevant to the study participants were selected from these validated frameworks. Informed consent was taken from the study subjects in the Hindi language. The questionnaire was administered through face-to-face interviews conducted by the researcher, who recorded the responses based on the participants’ answers to each question. It took six domains of QOL into consideration: physical, psychological, level of independence, social relations, environmental and spiritual/religious and personal beliefs. It consisted of 47 questions and the mean score of items within each domain was used to calculate the domain scores. A self-designed, semi-structured and pretested proforma was used to assess economic burden of PLWHA. Ethical approval of the study was obtained from the Institutional Ethics Committee, Rajendra Institute of Medical Sciences, Ranchi. Written informed consent of all the participants was taken.

The data were analysed by using IBM SPSS version 20.0 (IBM Corp., Armonk, NY). Frequency tables were generated to understand the distribution of variables. Student’s t-distribution test was performed to assess the association between the mean score of QOL and dichotomous variables. One-way analysis of variance (ANOVA) was used for the variables with multiple categories (e.g., age group, religion, marital status, educational status, socio-economic status, and CD4 cell count). Correlation was done to ascertain any relationship between continuous variables and stepwise multiple linear regression was performed to control the confounding effects of covariates and to understand the association between economic burden and QOL. For all statistical analyses, p<0.05 was considered significant.

## Results

Socio-demographic characteristics of the PLWHA 

The mean age (±standard deviation (SD)) of 216 patients was 40.48 (±11.87) years (Table 1). Among all the subjects, the majority (76, 35.2%) belonged to the age group 36-45 years, followed by people from the 46-55 years age group (52, 24.1%). More than half (53%) of the study subjects, that is, 114 were men. Most of participants come from a nuclear family (144, 72%) and were illiterate (73, 33.80%) with no formal education. Nearly half (112, 51.9%) of the study subjects were single followed by those who were married (53, 24.5%). Around 22.7% (49) were educated till the middle school followed by those who were educated till class 10 (40, 18.5%).

**Table 1:** Socio-demographic profile of people living with HIV/AIDS.

S. No	Socio-Demographic Profile	Variables	Frequency (N=216)	Percentage (%)
1	Age Group	18-25	33	15.30
		26-35	43	19.90
		36-45	76	35.20
		46-55	52	24.10
		≥56	12	5.60
2	Gender	Male	114	53.00
		Female	102	47.00
3	Religion	Hindu	153	70.80
		Muslim	27	8.79
		Sikh	2	0.92
		Christian	15	6.94
		Sarna	19	12.50
4	Ethnicity	Tribal	44	20.40
		Non-Tribal	172	79.60
5	Type Of Family	Nuclear Family	144	66.70
		Joint Family	72	33.30
6	Education	Illiterate	73	33.80
		Literate But No Formal Education	24	11.10
		Upto Primary Class	27	12.50
		Upto Middle School	49	22.70
		Upto Class 10	40	18.50
		Upto Class 12	1	0.50
		Graduation and above	2	0.90
7	Marital Status	Single	112	51.90
		Married	53	24.50
		Separated	3	1.40
		Widowed	41	19.00
		Divorced	7	3.20
		Single	112	51.90
8	Occupation	Daily Wage Worker	30	13.90
		Government Job	41	19.00
		Private Job	19	8.80
		Business	15	6.90
		Students	18	8.30
		Home Maker	16	7.40
		Truck/ Autodriver	66	30.60
		Unemployed	11	5.10
9	Residence	Rural	133	16.57
		Urban	83	38.43
10	Socio-Economic Status (Acd to Modified B G Prasad Classification 2020)	Class I	72	33.30
		Class II	29	13.40
		Class III	75	34.70
		Class IV	25	11.60
		Class V	15	6.90
11	CD4 Level	<200/mm^3^	104	48.16
		200-400/mm^3^	56	25.92
		> 400/mm^3^	56	25.92
12	Funding Received	No	151	69.91
		0-6 months	25	11.57
		7-12 months	40	14.82

Among the study subjects, 30.6% (66) were truck/auto drivers and were self-employed. The least common were students (18, 8.3%), 18 were homemakers (7.4%) and 11 were unemployed 11(5.1%). Most of the patients (133, 61.57%) belonged to the rural population and the rest (83, 38.43%) were from urban residential area. A majority of the study subjects (75, 34.7%) belonged to the Socio-economic Class III (INR 2260-7531 per capita per month). Regarding the CD4 level, 48.16% had CD4 level >400/mm^3^ and majority of the study subjects (151, 69.91%) did not receive funding. Within the study subjects who received the funding, 25 (11.57%) received funding for less than six months and the rest received the funding amount for more than six months.

QOL domains and economic burden of the study participants 

In this study, participants reported a mean QOL score (±SD) of 55.48 (±8.006) for the physical domain, 64.87 (±6.920) for the psychological domain, 54.95 (±5.308) for the level of independence domain, 60.67 (±5.586) for the social relationship domain, 67.98 (±3.940) for the environmental domain and 49.42 (±5.202) for the spiritual/religious/personal beliefs (SRPB) domain. The total QOL score was found to be 57.79 (±3.248) (Table 2). The mean total medical costs per month were found to be INR 22,942 (±4261.613) and the mean total non-medical costs per month amounted to INR 5342.97 (±382.367). Thus, the mean total direct costs per month were INR 23,593.94 (±4643.98). The indirect costs were calculated by taking into account the per capita GDP of India (INR 428/day). The average days lost due to being absent from work was 31.75 (±2.25). Adding both the direct and indirect costs, the total mean economic burden came out to be INR 36,182.94 (±5606.98) per month. 

**Table 2:** Quality of Life (QOL) Scores

Quality of Life Domain	Mean Score	Standard Deviation
Physical Domain	55.48	8.01
Psychological Domain	64.87	6.92
Level of Independence	54.95	5.31
Social Relations	60.67	5.59
Environmental Domain	67.98	3.94
Spiritual/ Religious/ Personal Beliefs Domain	49.42	5.20
Total QOL	57.79	3.25

Predictors of QOL 

Table 3 shows the association between the socio-demographic variables and the QOL. The younger age group of 18-25 years (p-value=0.001) coming from non-tribal background (p-value=0.001) and those who were literate but with no formal education (p-value=0.005) had significantly lower QOL.

**Table 3:** Association between socio-demographic variables and mean scores of six domains of QOL among PLWHA * denotes (p-value<0.05)- significant association. The tests used here are student t-test and analysis of variance (ANOVA). SRBP: Spiritual/religious/personal beliefs domain; PLWHA: people living with HIV and AIDS; SES: socioeconomic status.

Socio-Demographic Profile	Variables	Physical Mean (SD)	Psychological Mean (SD)	Level Of Independence Mean (SD)	Social Relationship Mean (SD)	Environment Mean (SD)	SRBP Mean (SD)	Total QOL Mean (SD)
Age Group	18-25 years	45.71 (8.32)	50.18 (5.90)	53.73 (6.22)	49.64 (4.43)	58.98 (2.99)	50.15 (4.66)	50.48 (2.52)
	26-35 years	55.65 (8.38)	54.98 (6.85)	53.95 (7.36)	51.34 (5.72)	61.19 (3.64)	50.31 (4.71)	53.48 (2.97)
	35-45 years	53.25 (8.95)	54.56 (6.86)	52.35 (7.06)	50.26 (5.85)	60.51 (3.61)	50.33 (4.79)	52.89 (3.34)
	46-55 years	53.80 (8.38)	54.74 (6.89)	53.44 (7.67)	50.76 (5.85)	60.55 (3.61)	50.33 (4.79)	52.89 (3.34)
P-value		0.000*	0.013*	0.680	0.705	0.101	0.959	0.001*
Gender	Male	55.52 (9.79)	64.44 (8.71)	54.78 (7.83)	60.93 (5.74)	75.85 (4.13)	56.64 (5.22)	62.80 (3.94)
	Female	55.46 (9.47)	65.23 (7.93)	55.08 (6.82)	60.43 (7.47)	69.56 (4.25)	56.32 (5.19)	63.26 (3.94)
P-value		0.980	0.484	0.773	0.570	0.000*	0.641	0.471
Religion	Hindu	52.07 (9.55)	53.54 (7.05)	52.88 (7.37)	50.66 (5.68)	60.58 (3.43)	50.06 (4.52)	52.31 (3.34)
	Muslim	54.13 (7.62)	55.26 (6.33)	50.67 (7.17)	50.22 (5.37)	59.32 (4.03)	49.41 (4.04)	52.86 (3.26)
	Sikh	64.00 (0.00)	56.00 (0.00)	48.00 (0.00)	48.00 (0.00)	56.89 (0.00)	49.78 (0.00)	52.67 (0.00)
	Christian	57.76 (3.39)	58.80 (5.06)	52.98 (4.02)	50.54 (5.83)	61.51 (4.02)	50.72 (5.45)	54.31 (2.64)
	Sarna	53.62 (8.68)	53.24 (6.66)	55.38 (5.13)	50.16 (5.26)	59.22 (2.49)	52.52 (5.74)	52.92 (2.31)
P-value		0.600	0.610	0.231	0.962	0.119	0.386	0.231
Ethnicity	Tribal	55.95 (7.15)	56.60 (6.14)	55.47 (5.99)	50.90 (5.16)	60.69 (3.31)	50.86 (4.47)	53.92 (2.87)
	Non-Tribal	52.05 (9.44)	53.40 (6.97)	52.72 (7.24)	50.46 (5.70)	60.36 (3.55)	50.03 (4.66)	52.18 (3.25)
P-value		0.011*	0.006*	0.011*	0.620	0.584	0.282	0.001*
Residence	Rural	54.40 (8.24)	64.17 (7.68)	55.08 (6.91)	54.54 (6.52)	68.55 (7.82)	56.46 (7.47)	59.89 (7.55)
	Urban	57.22 (7.89)	65.97 (7.00)	54.80 (6.44)	59.28 (6.22)	73.54 (7.37)	56.48 (7.74)	58.24 (7.53)
P-value		0.036*	0.118	0.830	0.016*	0.001*	0.983	0.992
Education Status	Illiterate	54.60 (10.24)	64.512 (5.99)	56.73 (8.44)	64.39 (4.96)	60.49 (3.10)	58.58 (4.19)	52.08 (3.43)
	Literate but no formal education	51.28 (6.54)	61.51 (7.37)	51.12 (5.71)	59.28 (6.31)	59.52 (3.59)	50.22 (5.63)	43.09 (4.20)
	Primary Education	57.64 (9.80)	68.42 (8.83)	55.60 (7.80)	60.98 (5.83)	60.17 (5.07)	55.8 (5.56)	50.75 (4.20)
	Middle Class	57.55 (7.49)	63.55 (5.86)	57.01 (5.29)	61.68 (4.81)	61.10 (3.32)	55.76 (4.07)	53.47 (2.27)
	Upto 10th Class	55.60 (9.01)	62.4 (6.57)	56.75 (5.05)	57.60 (4.39)	60.67 (3.02)	64.00 (4.41)	52.95 (2.56)
	Upto 12th Class	64.80 (0.00)	79.2 (0.00)	66.00 (0.00)	62.22 (0.00)	56.89 (0.00)	50.00 (0.00)	54.00 (0.00)
	Graduation and above	64.90 (0.00)	64.51 (0.00)	67.73 (0.00)	64.39 (0.00)	64.39 (0.00)	58.58 (0.00)	56.33 (0.00)
P-value		0.038*	0.001*	0.003*	0.039	0.120	0.083	0.005*
Marital Status	Single	54.67 (8.74)	54.08 (6.18)	52.32 (7.46)	50.84 (5.82)	60.53 (7.62)	50.04 (5.82)	52.72 (5.48)
	Married	51.74 (8.11)	54.64 (6.04)	57.2 (5.92)	50.46 (5.33)	59.30 (5.66)	50.27 (5.43)	52.76 (6.88)
	Separated	51.81 (7.98)	58 (7.47)	60.45 (7.38)	54.66 (6.29)	57.47 (5.90)	57.47 (6.73)	55.44 (4.36)
	Widowed	49.34 (7.45)	51.7 (7.53)	50.13 (7.14)	50.74 (5.40)	61.61 (6.95)	50.43 (4.22)	51.54 (6.00)
	Divorced	52.89 (9.23)	61.14 (8.25)	53.71 (8.99)	44.00 (4.31)	61.45 (7.78)	47.48 (5.81)	52.4 (6.34)
P-value		0.022*	0.008*	0.000*	0.019*	0.010*	0.038*	0.139
Socioeconomic Status	Class I	54.88 (8.21)	54.80 (6.45)	55.97 (6.82)	50.66 (4.65)	60.14 (3.14)	49.58 (3.60)	52.90 (2.45)
	Class II	53.51 (9.04)	54.58 (7.83)	53.68 (6.14)	50.40 (6.36)	61.31 (3.86)	49.97 (4.34)	52.88 (3.48)
	Class III	52.09 (8.58)	51.86 (5.60)	53.70 (7.76)	50.96 (6.06)	58.61 (3.06)	51.30 (6.15)	52.06 (3.04)
	Class IV	47.63 (10.04)	53.52 (7.26)	48.00 (8.98)	49.60 (4.97)	59.88 (2.84)	51.34 (5.46)	50.98 (4.22)
	Class V	49.82 (10.41)	52.94 (5.55)	53.81 (3.83)	51.60 (6.06)	61.74 (3.59)	50.26 (5.39)	52.67 (3.53)
P-value		0.007*	0.316	0.002*	0.830	0.003*	0.323	0.087
Occupation	Daily Wages Worker	51.20 (8.56)	63.67 (7.76)	53.57 (5.67)	62.47 (4.99)	74.42 (2.29)	55.00 (3.78)	63.93 (3.13)
	Government Job	58.41 (7.69)	69.48 (6.37)	53.13 (7.77)	60.33 (4.65)	74.58 (4.09)	56.14 (4.74)	63.79 (2.68)
	Private Job	59.79 (10.96)	63.40 (6.41)	58.13 (4.67)	60.76 (6.89)	77.41 (2.38)	58.52 (5.55)	62.4 (3.75)
	Owing a Business	54.57 (9.40)	63.84 (7.85)	53.54 (8.17)	60.00 (4.14)	73.63 (3.14)	58.80 (6.37)	61.68 (3.16)
	Student	55.05 (8.82)	60.93 (4.81)	53.92 (9.52)	59.59 (5.58)	75.74 (4.40)	58.22 (5.42)	61.8 (3.88)
	Homemaker	54.28 (8.29)	66.31 (6.73)	60.66 (7.92)	61.51 (5.41)	75.48 (3.02)	56.50 (3.92)	64.20 (3.05)
	Truck Driver	50.23 (8.56)	64.44 (6.85)	49.50 (5.10)	59.71 (6.19)	65.18 (3.19)	55.82 (4.11)	62.64 (3.12)
	Unemployed	55.84 (12.64)	61.75 (4.74)	57.50 (6.07)	62.61 (6.35)	75.59 (4.36)	56.00 (2.87)	63.72 (4.15)
P-value		0.019*	0.004*	0.000*	0.667	0.002*	0.108	0.205
CD4 Level	>400/mm^3^	59.11 (5.13)	65.66 (7.18)	65.66 (4.06)	60.48 (4.53)	74.29 (4.82)	56.52 (5.50)	62.56 (4.64)
	200-400	57.26 (4.28)	65.49 (4.35)	65.49 (4.71)	60.09 (4.43)	77.50 (4.23)	56.36 (3.84)	63.86 (4.11)
	<200	52.58 (5.64)	62.78 (5.81)	62.78 (5.36)	61.58 (5.99)	72.95 (5.68)	56.50 (3.75)	63.10 (4.23)
P-value		0.000*	0.092	0.947	0.463	0.000*	0.982	0.140

A correlation matrix showing correlation coefficients between variables is shown in Table 4. Each cell in the table shows the correlation between two variables. A strong positive correlation exists between the physical domain and psychological domain, level of independence, social relations domain, environmental domain and the total mean QOL scores, while economic burden shows a strong negative correlation with the physical domain. In the case of psychological domain, a positive correlation is observed with the level of independence domain and SRPB domain, and a strong positive correlation was seen between the psychological domain and social relations, environmental domain and total QOL score. Lastly, it was found that economic burden had a strong negative correlation with the physical domain, SRPB domain and the total QOL score. Table 5 highlights multiple linear regression analysis, showing a significant association between the independent variables total medical costs (p-value=0.022), loss of wages (p-value=0.005) and total economic burden (p-value=0.004) and the total QOL scores. The negative regression coefficient for all the three variables shows that there is a negative association, which can be interpreted that with the increase in the variables - total medical costs, loss of wages and total economic burden - there will be a significant decrease in the total QOL score. On the other hand, there was no association between total non-medical costs and total QOL.

**Table 4:** Correlation matrix between QOL domains and economic burden QOL: Quality of life; SRPB: spiritual/religious/personal beliefs domain; *significant correlation; **strong significant correlation.

Domains of QOL and Economic Burden	Physical Domain	Psychological Domain	Level of Independence	Social Relations	Environmental Domain	SRPB	Total QOL	Economic Burden
Physical Domain	1							
Psychological Domain	0.489* (0.000)	1						
Level of Independence	0.477** (0.000)	0.174* (0.011)	1					
Social Relations	0.231** (0.001)	0.186* (0.006)	0.255** (0.000)	1				
Environmental Domain	0.190** (0.005)	0.247* (0.000)	0.171* (0.012)	0.001 (0.985)	1			
SRPB	0.079 (0.247)	0.169* (0.013)	0.079 (0.250)	0.42 (0.544)	0.081 (0.234)	1		
Total QOL	0.797** (0.000)	0.661* (0.000)	0.616** (0.000)	0.528** (0.000)	0.393** (0.000)	0.148* (0.029)	1	
Economic Burden	-0.208** (0.002)	-0.028 (0.685)	-0.046 (0.505)	-0.061 (0.371)	-0.110 (0.108)	-0.176** (0.090)	-0.195** (0.004)	1

**Table 5:** Association between QOL among PLWHA and variables of economic burden QOL: Quality of life; * p-value<0.05 showing significant association; SE: standard error.

QOL Domain	Variables	Unstandardized Coefficients		Standardized Coefficients	t	p-value
		B	SE			
Total QOL	Total Medical Costs	-6.867	0.129	-0.156	-2.313	0.022*
	Total Non-Medical Costs	3.360	0.147	0.130	0.190	0.849
	Loss of Wages	-1.689	0.135	-1.910	-2.849	0.005*
	Total Economic Burden	-6.677	0.145	-0.195	-2.901	0.004*

## Discussion

This study revealed that PLWHA belonging to the younger age group of 18-25 years (p-value=0.001), coming from non-tribal background (p-value=0.001) and those who were literate but had no formal education (p-value=0.005) had significantly lower QOL. The reason behind this might be that being affected at such an age with a life-threatening disease, their willingness to live has been affected. Other socio-demographic variables like female gender, rural residence, widowed, participants belonging to modified BG Prasad Class IV and those working as truck drivers also showed lower QOL, but no significant association was seen. However, most of them showed a significant difference in one or more quality of life domains rather than on the total quality of life. In an in-depth analysis done by Lamkang and Aggarwal in Manipur, it was found that education and age did not have much effect on QOL outcomes, which is quite contradictory to our study [20]. The reason behind this maybe that this study had a majority of the people in the age group 31 and above (77%) and age group was categorized broadly. Our study corroborates with a study done in North India by Wig et al., which showed a significant difference in QOL scores with respect to the educational status (p<0.037) [13].

In this study, participants reported a total QOL score of 57.79±3.248 and the mean scores in different domains were highest in the environmental domain, with a score of 67.98 (±3.94) and lowest in the SRPB domain with a score of 49.42 (±5.20) followed by the level of independence domain with a score of 54.95 (±5.31). In a similar study by Folasire et al., the mean QOL scores on the scale of (0-100) in three domains were similar: psychological health (71.60±18.40); physical health (71.60±13.90), and the environmental domain (70.10±12.00), with the lowest score in the social domain (68.89±16.70) [21]. While in a study by Sarkar et al., the psychological domain (63.1±8.7) achieved the highest mean score followed by physical health with score of 56.2 (±9.8) on a 0-100 scale, A good score in the physical domain in this study might be due to the better services provided at the ART Centre of Calcutta School of Tropical Medicine (CSTM), Kolkata, which is one of the certified centres of excellence (COE) in HIV care in India [22]. However, the difference in our study and the given studies might be due to the fact that HIV/ AIDs patients are indifferent towards the environmental domain and so they are least affected by it, while the patients tend to lose their spiritual and religious beliefs when affected by any such life-threatening diseases. In another study by Rajeev et al., the QOL scores for all domains were intermediate for the PLHAs (10-14). The mean scores were highest for the psychological domain [23], thus showing the greater psychological impact on HIV/AIDS patients due to the disease. However, in the present study, the patient had a better mean score in the psychological domain, which might be due to better counselling, better healthcare services and ample support from their family members. At the ART centres in Jharkhand, several best practices are implemented to support the psychological well-being of people living with HIV, which likely contributes to the better mean scores observed in the psychological domain. Comprehensive counselling services are a core component; trained counsellors provide continuous psychosocial support, including individualized and family counselling sessions at different stages of treatment (pre-ART, ART initiation, and follow-up), addressing stigma, mental health concerns, adherence and disclosure issues. The ART centres ensure regular mental health screening and, when needed, referrals to specialized psychiatric or psychological care are facilitated, often in collaboration with institutions such as the Central Institute of Psychiatry in Ranchi [24,25].

In a cross-sectional study done by Gebremichael et al. on HIV/AIDS patients receiving anti-retroviral therapy in public health facilities in West Shoa Zone, Western Ethiopia, it was found that rural residence and the female gender were significantly associated with QOL domains. Illiterate individuals reported significantly lower QOL in the level of independence (adjusted OR=2.5 (95%CI:1.3,4.2)), social (adjusted OR= 1.5 (95%CI:1.1,4.4)) and environmental QOL in both female and male respondents [26]. The results of this study were in concordance with the present study, which shows a significant association (p-value<0.001) between only the gender and environmental domain of QOL. The environmental mean score (69.57±4.250) in the female gender was found to be lower as compared to the other domains of QOL. Likewise, a significant association was seen between residence and the physical domain and social relationship domain and the environmental domain. The physical mean (54.41±8.243) in the rural residence was found to be lower compared to the people belonging to the urban population. Similarly, the mean social relationship and environmental domain scores of the patients from rural residence were 54.54 (±6.520) and 68.55 (±7.823), respectively, and was comparatively lower than the urban counter parts.

In a longitudinal study done by Duraisamy et al., conducted at YRGCARE, Chennai, the amount of financial burden on the families of PLWHA was documented. Although the overall treatment costs were relatively low at INR 4636 in a six-month reference period, the average burden of the cost of treatment in the predominantly low-income population was 49 percent of the total household income [27]. Very few studies have been done in India focusing on the economic burden of HIV/AIDS patients' treatment after ARV drugs were given free of cost. But these studies have not included the costs before treatment such as diagnostic costs, private doctors' consultation costs, travelling costs etc. and still there are no studies showing an association between economic burden and QOL of HIV/AIDS patients. The negative regression coefficients as shown in the correlation matrix (Table 4) suggest that there is a negative association, which can be interpreted that with the increase in the variables of total economic burden, there will be a significant decrease in the total QOL score. Thus, there is a dire need for the government to pay heed to lessen this economic burden among the HIV/AIDS patients. The irregularities in the funding by the government add to these problems. The strength of this study lies in its pioneering nature as the first study to quantify the economic burden faced by PLHIV in Jharkhand. Additionally, the study provides valuable insights into QOL experienced by this population within the regional context. The use of validated and adapted QOL assessment tools enhances the reliability of findings. The limitations of this study are that it may be subjected to selection bias since the study subjects were the ones who were actively seeking routine medical care from health facilities. Those patients who do not come to the institutions from the communities may not be represented well in the study, therefore making this study generalizable only for those who are on ART follow-up. Many other social factors like social stigma and discrimination were not included, which may alter the QOL of PLWHA and might have acted as confounding factors in the study. The cross-sectional nature of the study itself limits conclusions on QOL over a period of time and difficult-to-know cause-effect temporal relationships could not establish the circumstances resulting in low QOL. Since only 216 HIV/AIDs patients were interviewed and the sample size taken was less than that required in the study, further studies should be done with a larger sample size.

## Conclusions

Economic burden thus plays an important part in affecting the lives of HIV/AIDS. According to a report by UNAIDS, poor people tend to be more vulnerable for a variety of reasons, including exposure to high-risk behaviours and no proper access to health services. HIV/AIDS may worsen poverty by reducing household income through loss of productivity, increased healthcare expenses and the need for caregiving. In developing countries like India, there is very little social security. This leads to a severe economic impact on HIV-affected families in developing countries as compared to developed countries. Though there are various factors like education, employment and stigma, which have led to poorer QOL, one cannot ignore the impact of financial insecurity on their life. There must be some financial intervention and rehabilitation programs catering to the needs of PLWHA, which will not only give them a sense of security but also lead them to have a community where they will feel welcome and be satisfied with their life.
